# Total outflow facility before and after goniotomy in *ex vivo* perfusion models for aqueous humor dynamics: effect of periocular tissue

**DOI:** 10.3389/fmed.2025.1705023

**Published:** 2026-01-12

**Authors:** Martin Kallab, Silvia Kaltenboeck, Parsa Panahi, Sarah Hinterberger, Matthias Bolz, Alex S. Huang, Clemens A. Strohmaier

**Affiliations:** 1 Department of Ophthalmology and Optometry, Kepler University Hospital, Johannes Kepler University, Linz, Austria; 2 Hamilton Glaucoma Center, The Viterbi Family Department of Ophthalmology, Shiley Eye Institute, University of California, San Diego, San Diego, CA, United States

**Keywords:** aqueous humor dynamics, distal outflow, glaucoma, minimally invasive glaucoma surgery (MIGS), porcine model

## Abstract

**Background:**

*Ex vivo* perfusion models to simulate aqueous humor dynamics are commonly used to test interventions for glaucoma treatment. Many models, however, overestimate the effect of surgical interventions. Periorbital tissue is routinely removed during the experimental preparation. Evidence suggests that up to 50% of total outflow resistance is attributable to the distal outflow pathways. It is currently unclear if varying degrees of tissue removal alone elicit changes in total outflow facility (C_tot_). We compared C_tot_ in whole globes with and without preserved periorbital tissue with intact trabecular meshwork (TM) and with surgical TM bypass in an *ex vivo* perfusion model.

**Methods:**

A total of 33 post-mortem porcine eyes with intact surrounding tissue were either trimmed (TISS−, *n* = 17) or left unchanged (TISS+, *n* = 16). Constant-flow perfusion at 4.5 μL/min and IOP measurement in the anterior chamber were performed. In a subgroup of 13 globes, a 5 mm goniotomy was performed before perfusion (7 TISS+, 6 TISS−). C_tot_ was analyzed once a stable equilibrium was reached.

**Results:**

C_tot_ was 0.27 ± 0.06 with intact TM and 0.36 ± 0.11 μL/mmHg/min with goniotomy in TISS+ globes, as well as 0.36 ± 0.12 and 0.47 ± 0.02 μL/mmHg/min in TISS− globes. Both comparisons (TM intact/ goniotomy) between TISS+ and TISS− globes were statistically significant (TM intact: *p* = 0.044, goniotomy: *p* = 0.031).

**Conclusion:**

This study demonstrates the influence of distal outflow pathways on C_tot_ with intact TM and after goniotomy. Thus, tissue preparation is a potential confounder in *ex vivo* AHO perfusion setups and may contribute to the different effect sizes of TM bypass surgery between *ex vivo* and *in vivo* studies.

## Introduction

Intraocular pressure (IOP) is the most important risk factor for glaucoma, and IOP lowering is the mainstay of glaucoma treatment (1). The modified Goldmann equation expresses steady-state intraocular pressure (IOP) as the balance between aqueous humor production and outflow facility, with episcleral venous pressure (EVP) as an additive factor (2). The structures downstream of Schlemm’s canal (including EVP) are commonly termed ‘distal outflow structures’ and known to account for up to 50% of the total outflow facility (i.e., the combination of trabecular meshwork (TM) and distal outflow facility) (3, 4). For the study of aqueous humor dynamics, *ex vivo* models are frequently used, and a recent consensus paper guides the optimal usage of such models (5). An important shortcoming of *ex vivo* models, however, is the incomplete modelling of the distal outflow resistance: EVP is zero, and the periorbital tissue (conjunctiva, portions of the episcleral, as well as eyelids and periorbital fat) is trimmed during tissue preparation. In our own unpublished experience with aqueous angiography, after tissue removal, we can sometimes see direct and immediate tracer flow through the cut aqueous and episcleral vein after tracer application, indicating that normal tissue anatomy is disrupted. Hence, in the present study, we set out to investigate the effect of periorbital tissue (including conjunctival portions of the episclera, periorbital fat, and eyelids) on the total outflow facility (C_tot_) in a porcine *ex vivo* perfusion model with the TM intact and after trabecular bypass surgery (goniotomy). In order to do so, C_tot_ between eyes with intact periorbital tissue (TISS+) or trimmed periorbital tissue (TISS− group) was compared during whole globe perfusion with the TM intact and after goniotomy.

## Methods

**Figure 1:**
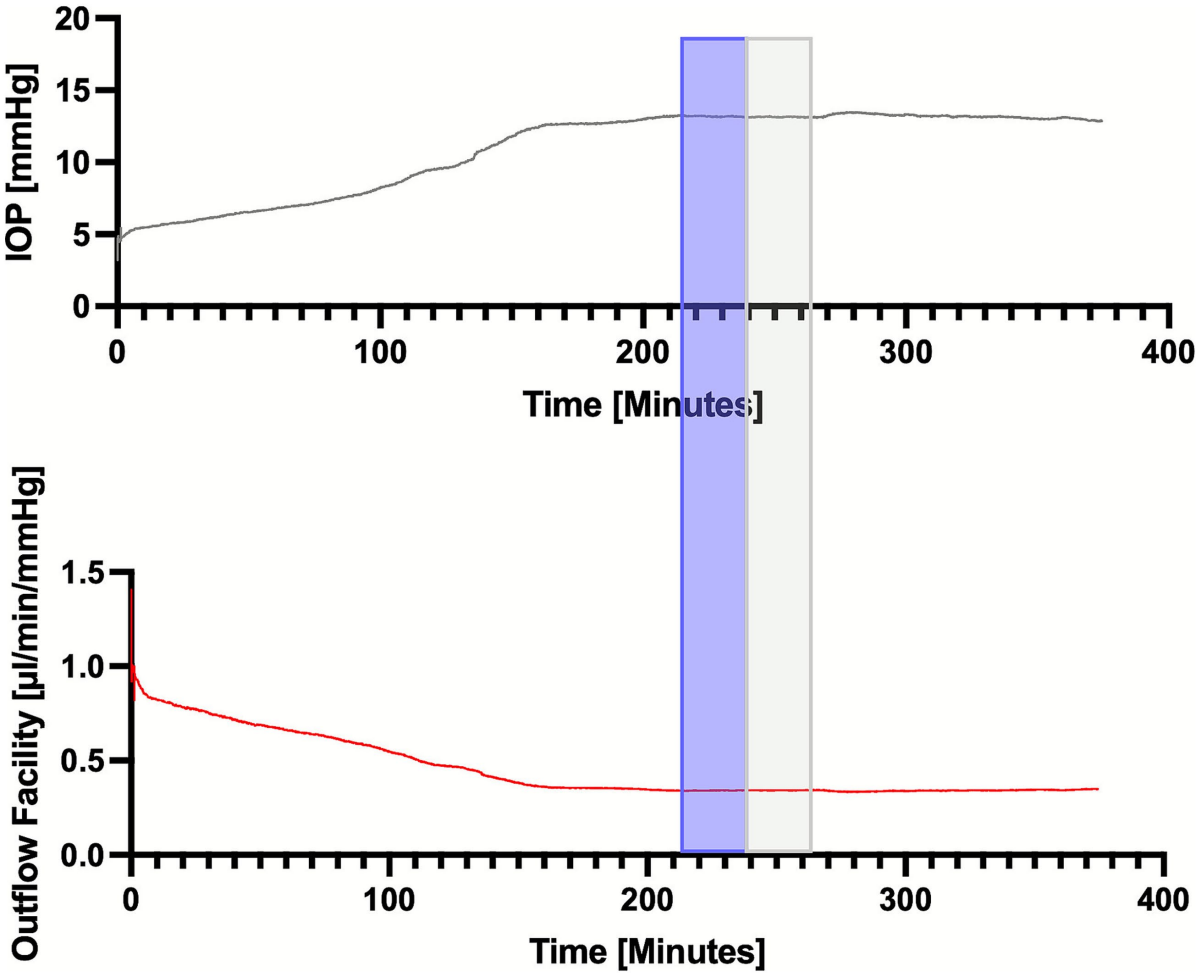
Representative single tracing of IOP (upper panel) and total outflow facility (lower panel) over time in a representative TISS+ globe with intact TM. Blue box: stability evaluation period and grey box: analyzed segment.

Thirty-three porcine eyes were acquired from a local abattoir immediately after the animals were sacrificed and transported to the laboratory. All eyes were stored in a moist environment at 4 °C and used within 24 h after collection. In the TISS+ group (*n* = 16), all orbital tissues were left intact, i.e., the conjunctiva, including the fornix, was intact (Supplementary Figure 1B). The medial and lateral canthus were cut gently to release tension and the weight of the eyelids from the bulbus, but the eyelids were otherwise left intact. In the TISS− group (*n* = 17), all surrounding ocular tissues were trimmed, and only a 5 mm conjunctival base was left at the limbus (Supplementary Figure 1A). After initial tissue preparation, both groups were handled using the same experimental protocol. A 25G needle was inserted into the anterior chamber and connected to a pressure transducer (MLT0380/A, ADInstruments Ltd., Oxford, United Kingdom) to measure IOP continuously. A second 25G needle was inserted into the anterior chamber parallel to the iris and positioned in the posterior chamber to simulate aqueous humor inflow (Supplementary Figure 1C). This needle was connected to a syringe pump (AL-1000, World Precision Instruments Germany, Friedberg, Germany). In 13 globes (7 TISS+, 6 TISS−), a needle goniotomy was performed as previously described (6). In short, globes were placed under a stereo microscope (OZM-922, Kern Optics, Balingen, Germany), and, via a paracentesis, a 25G needle was inserted. Using a surgical gonioscopy prism, the TM was visualized, and 5 mm cut was made with the needle tip. Afterward, the paracentesis was sealed using cyanoacrylate glue. In the experimental group with intact TM, the goniotomy was omitted. Perfusion was started with the flow rate set to 4.5 μL/min (at room temperature), and a stable equilibrium (defined as IOP change < 1 mmHg over 15 min) was awaited (5–8). Total outflow facility (C_tot_) was calculated by dividing the constant perfusion rate by the measured intraocular pressure average within a 20-min analysis period. Figure 1 illustrates the experimental protocol using a representative tracing: the grey bar highlights the stabilization period, and the blue bar the analyzed data segment. All data were recorded continuously using a digital data recording system (PowerLab C and LabChart 8, ADInstruments Ltd., Oxford, United Kingdom). Unpaired *t*-tests were used to compare C_tot_ values between TISS+ and TISS− globes (Prism GraphPad 10, GraphPad Software Inc., Boston, MA).

## Results

**Figure 2:**
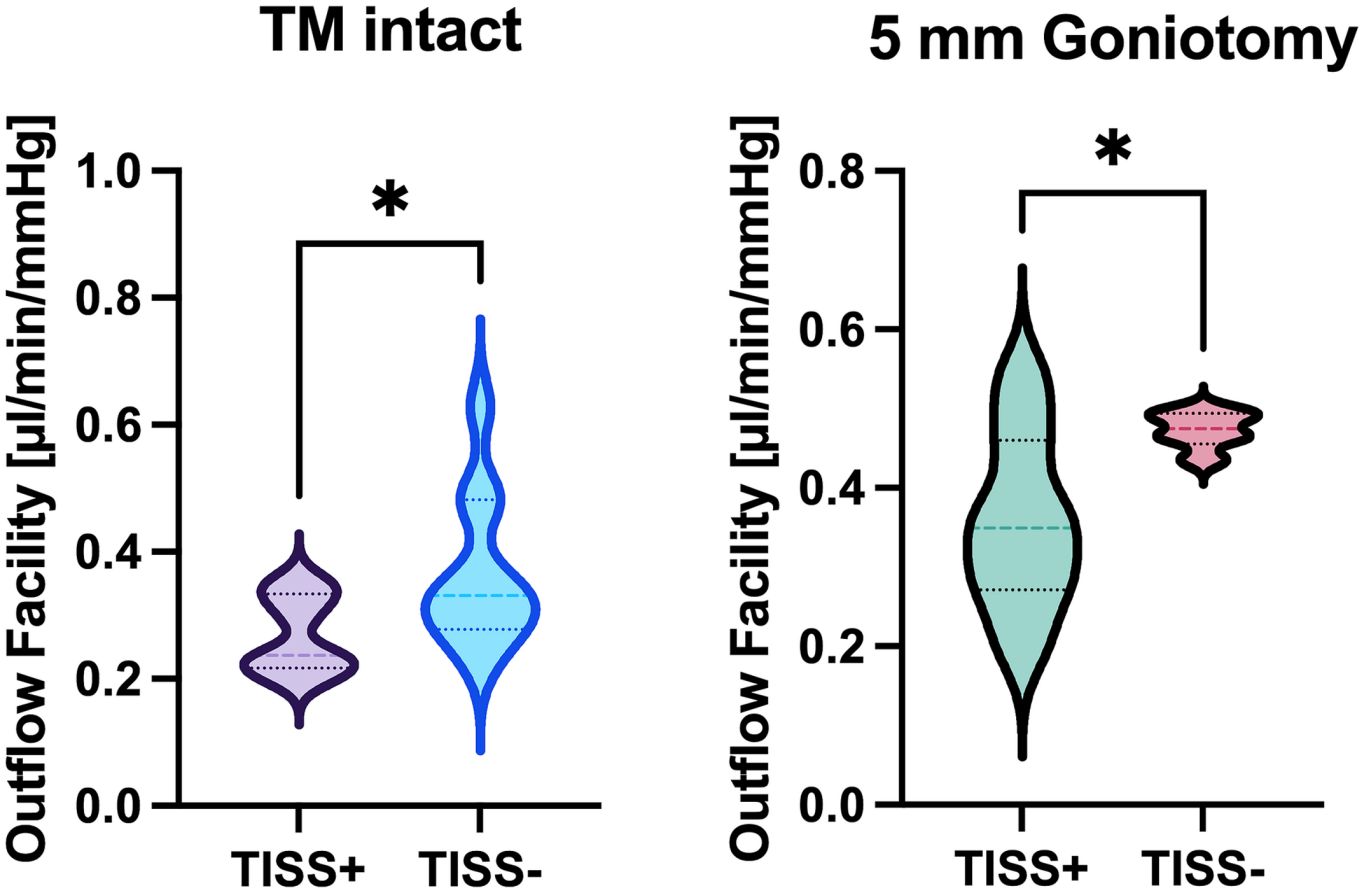
Total outflow facility comparison between TISS+ and TISS− globes with intact TM (left panel) and after goniotomy (right panel). *p < 0.05 for unpaired *t*-test.

In all eyes, a stable equilibrium was reached. With the TM intact, C_tot_ was 0.27 ± 0.06 μL/mmHg/min in TISS+ globes and 0.36 ± 0.12 μL/mmHg/min in TISS− globes. After surgical TM bypass, TISS+ globes showed a C_tot_ of 0.36 ± 0.11 μL/mmHg/min and TISS− globes a C_tot_ of 0.47 ± 0.02 μL/mmHg/min. As visualized in Figure 2, differences between TISS+ and TISS− were statistically significant for globes with intact TM (*p* = 0.0443) and for globes after TM bypass (*p* = 0.0310).

## Discussion

In this study, we report significant outflow facility differences between eyes with and without preserved periorbital tissue after trabecular bypass surgery and with intact TM. C_tot_ values in the TISS− groups are comparable to previously published outflow facilities in porcine anterior segment and whole globe perfusion setups, although previously reported values are subject to notable inter-study variability (4, 7, 9–22). The exact reason for the observed C_tot_ differences between TISS+ and TISS− globes is currently unknown, and research of involved anatomical structures has been proven to be intricate: A recent consensus statement on *ex vivo* AHO perfusion setups acknowledges the incomplete modelling of the distal outflow resistance after Schlemm’s canal (collector channels, scleral vessels, and episcleral circulation) as a significant model limitation (5). Although distal outflow resistance has been identified as a significant and pharmacologically modifiable contributor to total outflow resistance in porcine and human anterior segments with trimmed conjunctiva (4), the role of downstream anatomical structures, including the episcleral circulation in IOP regulation, is still unclear. The episcleral circulation has unique and partially unexplored features suggesting a role in IOP homeostasis: 1) lack of a capillary bed, 2) two distinct types of autonomously innervated arteriovenous anastomoses (AVAs) in multiple species (23–27), and 3) the capability of these AVAs to alter IOP and EVP upon pharmacological manipulation and/or neuronal stimulation in rabbits and rats (28–31). Owing to this complex vascular architecture, EVP measurement itself has yielded varying results over the years, depending on the species, location of measurement, and measurement method (31–33). Due to the lack of venous pressure in *ex vivo* models for AHO (another limitation stated in the above-mentioned consensus statement), the uncertainty concerning EVP has conventionally been irrelevant in these reductionist scenarios. By omitting EVP, the outflow facility can be calculated by dividing the rate of inflow by IOP in these models (2, 5). This also holds true for the calculation of the total outflow facility in TISS+ and TISS− globes in this report. To address the limitation of missing EVP, we recently presented an experimental approach to simulate above-zero EVP values by ophthalmic artery perfusion (34).

The effect size of intact periorbital tissue on total outflow facility (~30%) is comparable between globes with and without goniotomy. Of note, TISS− globes with intact TM and TISS+ eyes after goniotomy had a comparable mean C_tot_ of 0.36, suggesting a similar effect of intact conjunctiva/episcleral and intact TM on C_tot_—at least in our porcine whole globe perfusion setup. More experiments, including intraluminal pressure measurements along the outflow pathway, are necessary to precisely determine the mentioned effect sizes. In their current form, the findings of the present study indicate that the periorbital tissue and its imposed outflow resistance may be a confounder when *ex vivo* aqueous humor dynamics models are used.

We are aware that our results are of a preliminary character, and the setup used has shortcomings that need to be acknowledged. First of all, only relatively short-term perfusions have been performed. Furthermore, perfusion has been performed at room temperature. This is based on previous studies using a similar setup that allowed for measurements for up to 7 h before the well-known washout phenomenon set in, and this model has been used successfully for surgical interventions in the past (6, 8). A stable equilibrium could, however, be reached in all eyes, indicating the validity of the data for the observed time period, and no difference in the appearance of eyes between the groups was noted.

Moreover, we did not measure intraluminal pressures in episcleral vessels. While this information would undoubtedly be of high scientific interest, the complex architecture of the episcleral circulation and dependency on position relative to AVAs complicate such measurements. Finally, our current approach cannot answer which exact anatomical structures that were removed/compromised in the TISS− group are responsible for the observed C_tot_ differences (i.e., where the increased resistance in the TISS+ group is generated). Simultaneously, multipoint intraluminal pressure, as well as episcleral flow measurements combined with *in silico* simulations, might overcome the latter two limitations in the future (35).

In summary, this report highlights the influence of periorbital tissue integrity on outflow biology in *ex vivo* AHO perfusion setups. Therefore, researchers ought to be aware of tissue preparation as a potential confounder in studies involving these setups, especially when focusing on the characteristics of the distal outflow pathways. More studies are necessary to precisely characterize the exact anatomical structures in the periorbital tissue contributing most to the observed facility difference, to study the effect of intraluminal pressures, and to analyze potential regulation mechanisms.

## Data Availability

The raw data supporting the conclusions of this article will be made available by the authors, without undue reservation.

## Supplementary material

SUPPLEMENTARY FIGURE 1 Pictures of the experimental setup in the TISS− **(A)** and TISS+ **(B)** group. **(C)** A magnified view of the needle position in the anterior and posterior chamber.
