# Supplementary figures and images for: Total outflow facility before and after goniotomy in ex vivo perfusion models for aqueous humor dynamics: effect of periocular tissue
Source: Front Med (Lausanne). 2026 Jan 12;12:1705023. doi: 10.3389/fmed.2025.1705023 (PMC12832705; doi:10.3389/fmed.2025.1705023)

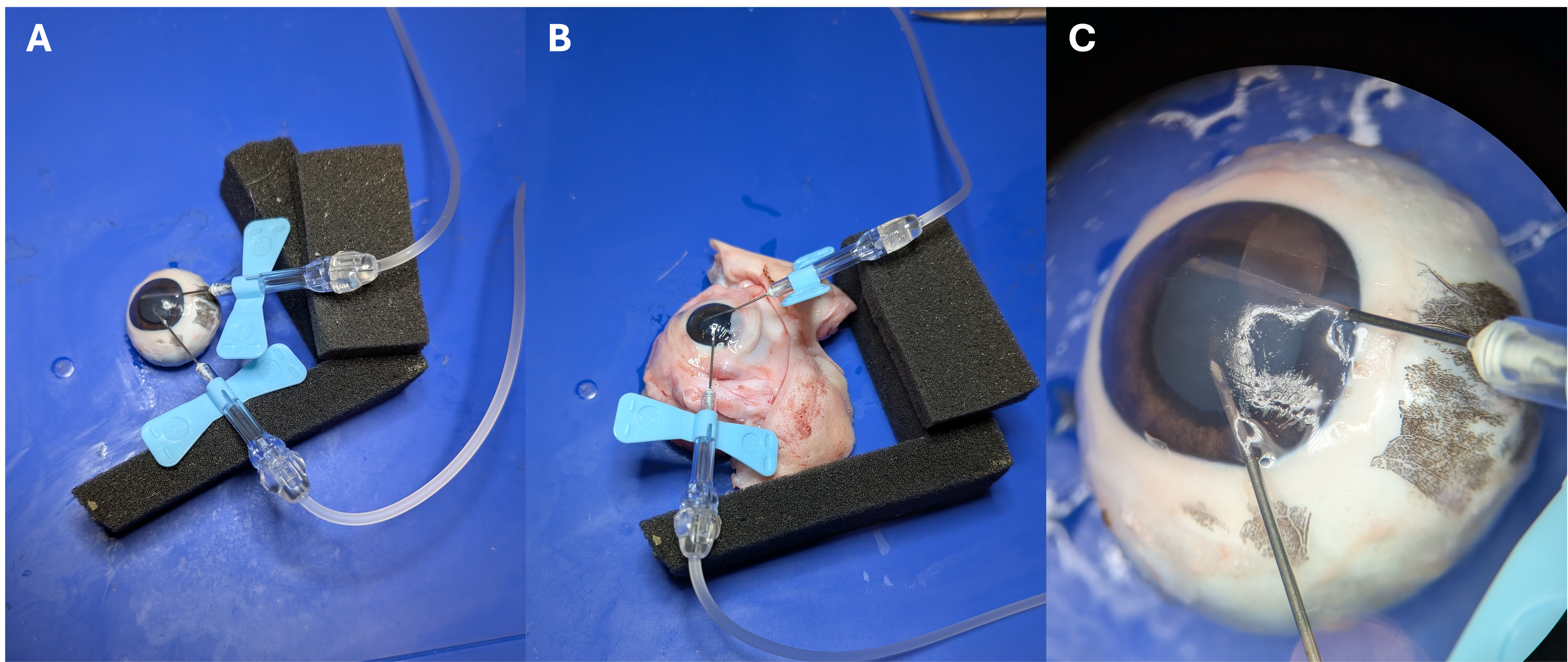

Supplement: SUPPLEMENTARY FIGURE 1 — Pictures of the experimental setup in the TISS− (A) and TISS+ (B) group. (C) A magnified view of the needle position in the anterior and posterior chamber. [file Image_1.TIFF]
